# Hepatitis C virus care continuum: integrating point of care RNA assay and direct acting antivirals

**DOI:** 10.17179/excli2024-7963

**Published:** 2025-01-21

**Authors:** Rabab Fatima

**Affiliations:** 1 Department of Chemistry, UPES, Dehradun 248007, India

## ⁯⁯⁯

Since its discovery in 1989, the hepatitis C virus (HCV), a hepatotropic RNA virus, has emerged as a global health threat with an estimated 71 million individuals infected worldwide, the vast majority of whom remain oblivious to their condition. This bloodborne virus exhibits profound genetic heterogeneity, spanning seven major genotypes and numerous subtypes. HCV primarily targets hepatocytes, leading to chronic infection in nearly 80 % of cases, particularly among people who inject drugs (PWID) or people in prison (Tariq et al., 2022[8]). The virus's transmission pathway, unsafe medical procedures, intravenous drug use, and pre-screening-era blood transfusions contribute to its epidemiological complexity. Common clinical manifestations include fatigue, anorexia, dark urine, jaundice, malaise, constipation and nausea. The protracted asymptomatic phase exacerbates undetected viral persistence, leading to severe hepatic complications such as cirrhosis and hepatocellular carcinoma. Epidemiological surveillance demonstrates critical HCV diagnostic disparities between low and middle-income countries (LMICs) (8 % case identification) and high-income nations (43 % diagnostic rates). Achieving WHO's HCV elimination targets necessitates implementation of decentralized diagnostic architectures utilizing point-of-care (POC) technologies, task-shifting frameworks, and integrated service delivery models, supported by tiered laboratory networks and simplified treatment protocols in resource-constrained settings (Oru et al., 2021[5]). 

Recent advancements in point-of-care RNA nucleic acid test viral load assays have redefined the clinical HCV landscape, offering superior precision at an accelerated pace (Grebely et al., 2024[1]). Conventional HCV diagnosis relied on a two-step protocol: initial serological antibody testing followed by a confirmatory RNA detection test, delaying definitive diagnosis by several days or weeks, often resulting in patient attrition. The emergence of highly sensitive and rapid POC HCV RNA assays has drastically optimized this workflow. With an ability to detect viral RNA at concentration as low as 10-15 IU/ml, TrueNat and GeneXpert leveraged real time and chip based RT PCR, to deliver results in 60-90 minutes further expediting clinical decision making (Liu et al., 2020[4]). This paradigm shift in diagnostics is crucial in resource stretched settings and hard to reach populations, where traditional laboratory infrastructure is inadequate or often unavailable. When paired with contemporary direct acting antiviral (DAA) regimen synergistically, a sustained virological response exceeding 95 % across all genotypes, a condensed treatment duration of 8-12 weeks along with a favorable safety profile was achieved. These therapies also precisely targeted non structural proteins such as NS5A and NS5B polymerases and NS3/4 A proteases, associated with viral replication (Zhang et al., 2023[9]). Pan genotypic DAA regimen utilizing Sobosbuvir/Velpatasvir and Glecaprevir/Pibrentasvir have further demonstrated a high genetic barrier to resistance, obviating the need for genotype specific therapy and simplifying the clinical management of this disease (Schiano Moriello et al., 2024[6]). The integration of rapid POC diagnostics with an effective DAA regimen, directly aligns with WHO's initiative to eradicate HCV, as a global health threat, by 2030.

POC HCV viral load quantification assays demonstrate robust analytical concordance with centralized laboratory methodologies, offering a clinically validated alternative for viremia confirmation and expedited therapeutic intervention across diverse clinical settings (Kapadia et al., 2024[3]). While marginally reduced analytical precision may be observed in POC implementations, the enhanced operational simplicity and improved patient care continuum metrics substantiate their clinical utility. Meta-analytical evidence from systematic reviews evaluating POC HCV viral load testing utilizing Xpert HCV Viral load, Genedrive, TrueNat, HCV ID Kit, SAMBA II HCV and Xpert HCV VL Fingerstick assay demonstrated statistically significant improvement in key performance indicators such as treatment initiation turnaround time (TAT) was reduced to 18.5 days [95 % CI: 14-53] with POC testing compared to 67 days [95 % CI: 50-67] for conventional laboratory-based high-throughput RNA assay. Furthermore, therapeutic uptake demonstrated marked enhancement, achieving 77 % [95 % CI: 72-83 %] compared to 53 % [95 % CI: 31-75 %] with traditional laboratory methodology (Tang et al., 2022[7]). This optimization of the diagnostic-therapeutic cascade exhibits particular significance in epidemiologically critical populations, notably in PWID characterized by elevated HCV prevalence and substantial attrition rates.

Integration of POC HCV RNA quantification with POC liver staging constitutes the optimal diagnostic protocol per WHO guidelines that recommend liver staging prior to treatment initiation. However, if the diagnostic and therapeutic algorithm excludes decompensated cirrhosis based on clinical signs, it may be acceptable to initiate treatment based solely on HCV RNA detection, while awaiting definitive liver staging assessment (Ivanova Reipold et al., 2024[2]). By enhancing diagnostic efficiency, reducing patient loss to follow-up, and enabling streamlined test-and-treat protocols, this advancement provides a critical vector for HCV micro elimination. The COVID-19 pandemic which has catalyzed accelerated development of ultra-sensitive, rapid, and cost-effective diagnostic technologies, hold promise to revolutionize HCV diagnostic procedures. Nevertheless, challenges in scaling these technologies, addressing healthcare heterogeneity, and overcoming socioeconomic barriers remain formidable. Despite these hurdles, the combined efficacy of rapid diagnostics and advanced therapeutics offers a definitive strategy for mitigating the global burden of HCV and related exigencies.

## Conflict of interest

The author declares no conflict of interest.
